# Menopausal Women With Diabetes and Comorbid Anxiety: Integrated Management Involving Cognitive Behavioral Therapy, Diabetes Education, and Pharmacotherapy

**DOI:** 10.7759/cureus.96251

**Published:** 2025-11-06

**Authors:** Aasim Mohammad Bhat, Shahid Shehzad, Sadia Chaudhary, Mohsin Ahmad, Sarfaraz Khan, Nayab Saad, Awais Hameed, Iftikhar Khattak, Harry Jia

**Affiliations:** 1 Medicine, Ali Imran Hospital, Rawalakot, PAK; 2 Department of Diabetes and Endocrinology, Bacha Khan Medical College, MTI-Mardan Medical Complex, Mardan, PAK; 3 Obstetrics and Gynecology, Rahbar Medical and Dental College, Lahore, PAK; 4 Neurology, Rai Medical College Teaching Hospital, sargodha, PAK; 5 Internal Medicine, Mercy Teaching Hospital, Peshawar Medical College, Peshawar, PAK; 6 Obstetrics and Gynecology, Russells Hall Hospital, Dudley, GBR; 7 Department of Bioinformatics and Biotechnology, Government College University, Faisalabad, PAK; 8 Department of Research and Development, Celestial and Dimanche, Muzaffarabad, PAK; 9 Faculty of Medicine, Hamdard University, Islamabad, PAK

**Keywords:** anxiety, cognitive behavioral therapy, depression, diabetes, hba1c, integrated management, machine learning, menopause

## Abstract

Background: Menopausal women with diabetes frequently experience comorbid anxiety, which complicates both metabolic and psychological management. Integrated interventions combining cognitive behavioral therapy (CBT), diabetes education, and pharmacotherapy may offer comprehensive benefits, yet evidence remains limited.

Methodology: A retrospective study was conducted among menopausal women with diabetes and anxiety. Participants were categorized into three groups: CBT + education + pharmacotherapy, education only, and medications only. Clinical, demographic, and psychological data were analyzed using descriptive and inferential statistics. Machine learning (ML) models, including Logistic Regression, Random Forest, and XGBoost, were applied to identify predictors of treatment outcomes, with SHAP analysis used for model interpretability.

Results: The study included 300 women with a mean age of 54.13 years and a body mass index (BMI) of 28.07 kg/m². HbA1c averaged 7.47% and fasting glucose 140.08 mg/dL. Socioeconomic distribution was high (*n* = 106, 35.3%), middle (*n* = 106, 35.3%), and low (*n* = 88, 29.3%). Blood pressure categories differed significantly across groups (χ² = 0.037, *P* < 0.05). Anxiety scores trended toward improvement under integrated management (*F* = 3.707, *P* = 0.055). Logistic regression highlighted age (Exp(*B*) = 1.045, *P* = 0.064) and cognitive function (Exp(*B*) = 1.087, *P* = 0.090) as borderline predictors. ML outperformed logistic regression, with Random Forest (accuracy 81.5%, Receiver Operating Characteristic-Area Under the Curve (ROC-AUC) 0.85) and XGBoost (accuracy 83.4%, ROC-AUC 0.82). Depression score (mean 11.80, standard deviation (SD) = 4.54), HbA1c, and treatment history emerged as key predictors.

Conclusions: Integrated management strategies appear to be effective in addressing both metabolic and psychological outcomes. Ensemble ML offers superior predictive insight, supporting personalized care.

## Introduction

Menopause is a natural biological transition that typically occurs between the ages of 45 and 55 and is characterized by the cessation of ovarian activity and a decline in estrogen and progesterone production. This hormonal shift contributes not only to vasomotor symptoms such as hot flashes, sleep disturbances, and night sweats, but also to an increased risk of chronic conditions, including cardiovascular disease, osteoporosis, and metabolic disorders [1,2]. Globally, more than one billion women are projected to be postmenopausal by 2025, with approximately 47 million women entering menopause each year [2]. Among this population, type 2 diabetes (T2D) is particularly prevalent, with estimates suggesting that 20%-30% of postmenopausal women are affected, mainly due to age-related metabolic changes and the loss of estrogen’s protective effects on glucose regulation [1,3]. At the same time, the burden of mental health disorders is rising; anxiety is reported in nearly 40% of menopausal women, and 16% of new cases are diagnosed during the postmenopausal period [4,5].

The coexistence of diabetes and anxiety during menopause represents a unique and complex challenge. Evidence shows that the relationship is bidirectional: poor glycemic control exacerbates anxiety through fear of complications, while anxiety undermines diabetes self-management, medication adherence, and lifestyle modification [6,7]. This interaction creates a vicious cycle, leading to higher rates of hospitalization, poorer glycemic outcomes, increased healthcare utilization, and reduced quality of life compared with women who have only one of these conditions [3,8]. Moreover, untreated anxiety in women with diabetes is associated with poor long-term outcomes, highlighting the urgent need for interventions that target both conditions simultaneously [5,9].

Traditional diabetes management in postmenopausal women emphasizes pharmacotherapy, diabetes education, and lifestyle interventions such as diet modification, exercise, and weight management. While these strategies have been successful in controlling blood glucose levels and preventing complications, they often fail to adequately address coexisting psychological symptoms [10,11].

Cognitive behavioral therapy (CBT), an evidence-based intervention for anxiety and depression, has been increasingly studied in both menopausal and diabetic populations. In menopausal women, CBT has proven effective in reducing anxiety, depression, and insomnia, and in improving coping with vasomotor and psychosomatic symptoms [12-14]. In patients with diabetes, CBT has demonstrated significant improvements in psychological well-being, treatment adherence, glycemic control, and overall quality of life [3,6,15]. When CBT is combined with diabetes self-management education and pharmacotherapy, outcomes are synergistically improved, with reductions in diabetes distress, enhanced metabolic stability, and better mental health [5,7].

Despite these promising findings, the literature remains fragmented. Many studies have evaluated CBT for menopausal anxiety or for diabetes distress in isolation, while others have examined diabetes education or pharmacological management independently. Few studies, however, have specifically assessed the combined effects of CBT, diabetes education, and pharmacotherapy in menopausal women with comorbid diabetes and anxiety [8,13,16]. This gap is particularly concerning given that over half of women with diabetes are likely to experience psychological distress during menopause [2]. Addressing this issue through integrative strategies is essential for improving adherence, reducing complications, and enhancing long-term well-being.

Therefore, this retrospective research seeks to evaluate the outcomes of an integrated management approach that combines CBT, diabetes education, and pharmacotherapy in menopausal women with diabetes and comorbid anxiety. This study aims to generate evidence supporting holistic, patient-centered care strategies that may improve treatment adherence, metabolic outcomes, and quality of life in this vulnerable and growing population by simultaneously targeting these conditions' physiological and psychological dimensions.

## Materials and methods

Study design, data source, and participant selection

A multi-center, retrospective cohort study was conducted using de-identified electronic health records (EHRs) obtained from collaborating tertiary-care hospitals and affiliated outpatient clinics under a data-use agreement and institutional review board (IRB) approval (waiver of consent for minimal-risk secondary analysis). From this network, adult women with documented type 2 diabetes (T2D) and menopausal status were identified if they had ≥1 clinical encounter during the study window. Inclusion criteria were defined as follows: (1) female sex, age ≥18 years; (2) T2D confirmed by International Classification of Diseases, 10th Revision (ICD-10) codes (E11.x), laboratory evidence (e.g., ≥2 HbA1c results ≥6.5% on separate dates), or active use of glucose-lowering medication; (3) menopause documented as natural or surgical in the EHR (or inferred by an age-based proxy with ≥12 months of amenorrhea when explicit documentation was unavailable); and (4) comorbid anxiety at or before the index date, defined by ICD-10 codes F40-F41 or a clinician diagnosis supported by a concordant anxiolytic or selective serotonin reuptake inhibitor (SSRI) prescription. Exclusion criteria were applied for pregnancy during the study window, active cancer therapy, conflicting sex records, or missing primary exposure/outcome variables after quality checks. The index date was assigned as the earliest encounter meeting all inclusion criteria. After application of these criteria, a final analytic cohort of 300 menopausal women with T2D and comorbid anxiety was assembled.

Missing data

Missingness was assessed, and a missing at random (MAR) mechanism was assumed. Covariates were imputed using multiple imputation by chained equations (MICE; *m* = 20) with predictive mean matching for continuous variables and logistic or polytomous regression for categorical variables, followed by pooling according to Rubin’s rules. Variables with ≥20% missingness were excluded from primary models. Implausible values were removed by clinical thresholds, and limited winsorization (0.5th/99.5th percentiles) was pre-specified to reduce undue leverage; robustness was verified via complete-case and no-winsorization sensitivity analyses.

Exploratory data analysis

Before statistical and predictive modeling, exploratory data analysis (EDA) was conducted to evaluate the quality and structure of the dataset. Missing values were assessed and handled using mean imputation for continuous variables and mode substitution for categorical variables. Outliers were detected through boxplots and z-scores, with extreme values in HbA1c and fasting glucose adjusted using winsorization. Distributional assumptions were tested using the Shapiro-Wilk test, and skewed variables were standardized. Correlations between clinical and psychological parameters were assessed using Pearson’s and Spearman’s coefficients to examine relationships such as HbA1c and anxiety. Visualization tools, including histograms, scatterplots, and heatmaps, were used to explore distributions and identify potential multicollinearity among variables.

Data analysis

Statistical analyses were conducted in IBM SPSS (v27.0) and Python (v3.0; pandas/numpy/statsmodels-scikit-learn). Descriptive statistics were generated, reporting mean ± SD (and ranges) for continuous variables and frequencies (*n* (%)) for categorical variables. Group differences in categorical variables-including comorbidities, blood-pressure categories, and treatment history-were assessed with χ² tests (Fisher’s exact when sparse). Continuous variables were compared using independent-samples t-tests or one-way ANOVA across intervention groups (e.g., CBT with diabetes education and pharmacotherapy vs. education only vs. pharmacotherapy only). Normality and homoscedasticity were assessed using the Shapiro-Wilk and Levene tests, respectively, and nonparametric alternatives (Wilcoxon/Mann-Whitney or Kruskal-Wallis) were applied when assumptions were violated. Predictors of positive primary outcomes were modeled using multivariable logistic regression with age, BMI, HbA1c, fasting glucose, depression scores, and comorbidities as independent variables; results were reported as adjusted odds ratios (aORs) with 95% confidence intervals (CIs) and two-sided *P*-values. For the multi-center design, site effects were accommodated using fixed effects with cluster-robust standard errors. Analyses were performed on multiply imputed datasets with estimates pooled via Rubin’s rules, and sensitivity analyses (complete-case and no-winsorization) were conducted to assess robustness; statistical significance was set at α = 0.05. Assumptions for parametric tests were verified before applying t-tests and analysis of variance (ANOVA). Normality was assessed using Shapiro-Wilk tests and Q-Q plots; homogeneity of variances was evaluated with Levene’s test (or the Brown-Forsythe test when appropriate); independence was ensured by study design; and outliers were identified using boxplots and studentized residuals. When assumptions were violated, Welch’s t-test/ANOVA or nonparametric alternatives (Mann-Whitney U, Kruskal-Wallis) were used, with Games-Howell or Dunn’s tests (as applicable) for post hoc comparisons. Where helpful, transformations were explored as sensitivity analyses, and effect sizes (Cohen’s *d*, η²/ω²) were reported alongside *P*-values.

Machine learning models

To complement traditional statistics, three supervised machine learning (ML) models were developed using Python in Google Colab. Logistic regression served as the baseline linear model, offering interpretable coefficients for clinical prediction. Random Forest, an ensemble of decision trees, was employed to capture non-linear interactions; feature importance analysis identified BMI, risk score index, fasting glucose, HbA1c, and anxiety score as dominant predictors. XGBoost, a gradient boosting algorithm optimized for structured health data, was also trained and revealed intervention type, depression score, treatment history, and HbA1c as critical predictors of outcomes. Together, these models allowed for both predictive accuracy and interpretability of results.

Variables and prediction approach

Independent variables included age, BMI, HbA1c, fasting glucose, blood pressure, anxiety and depression scores, risk index, comorbidities, treatment history, medications, socioeconomic status, cognitive function, and intervention type. The dependent variable was a binary outcome reflecting improvement (HbA1c reduction and/or anxiety reduction) versus no improvement. Preprocessing involved encoding categorical variables using label encoding and scaling continuous variables with StandardScaler. The dataset was split into training (80%) and testing (20%) subsets with stratification to preserve outcome proportions. To address class imbalance, oversampling using the Synthetic Minority Oversampling Technique (SMOTE) was applied. Model performance was evaluated using accuracy, precision, recall, F1-score, and Receiver Operating Characteristic-Area Under the Curve (ROC-AUC). A combined ROC curve was generated to visualize and compare the diagnostic performance of all three models.

Rationale for a dual approach (SPSS regression + ML)

A dual approach was used to pair clear inference with strong prediction. SPSS regression was applied to yield interpretable, adjusted effect estimates (ORs/β) with CIs and *P*-values for hypothesis testing and clinical interpretation. ML models in Python were trained to capture nonlinearity and interactions, optimizing discrimination and calibration via cross-validation. Agreement between variable importance and regression effects was assessed; any discrepancies were examined using diagnostic analyses. Regression models were used to explain relationships, while ML methods enhanced risk stratification, together improving overall robustness. Hyperparameter tuning was performed using nested cross-validation (inner CV for grid or random search; outer k-fold for generalization), with early stopping and regularization applied where appropriate. Final models were refit on training folds only and evaluated on an independent hold-out test set to prevent overfitting. Feature importance (permutation/SHAP) was computed exclusively on the independent test set using the frozen final model to avoid information leakage.

Ethical considerations

This study was conducted on anonymized secondary data and did not contain identifiable patient information. Institutional approval was obtained, and the research complied with the principles of the Declaration of Helsinki. Data protection and confidentiality standards were maintained throughout. The ML models developed were intended solely for research purposes and not for clinical application.

## Results

Descriptive statistics

**Figure 1:**
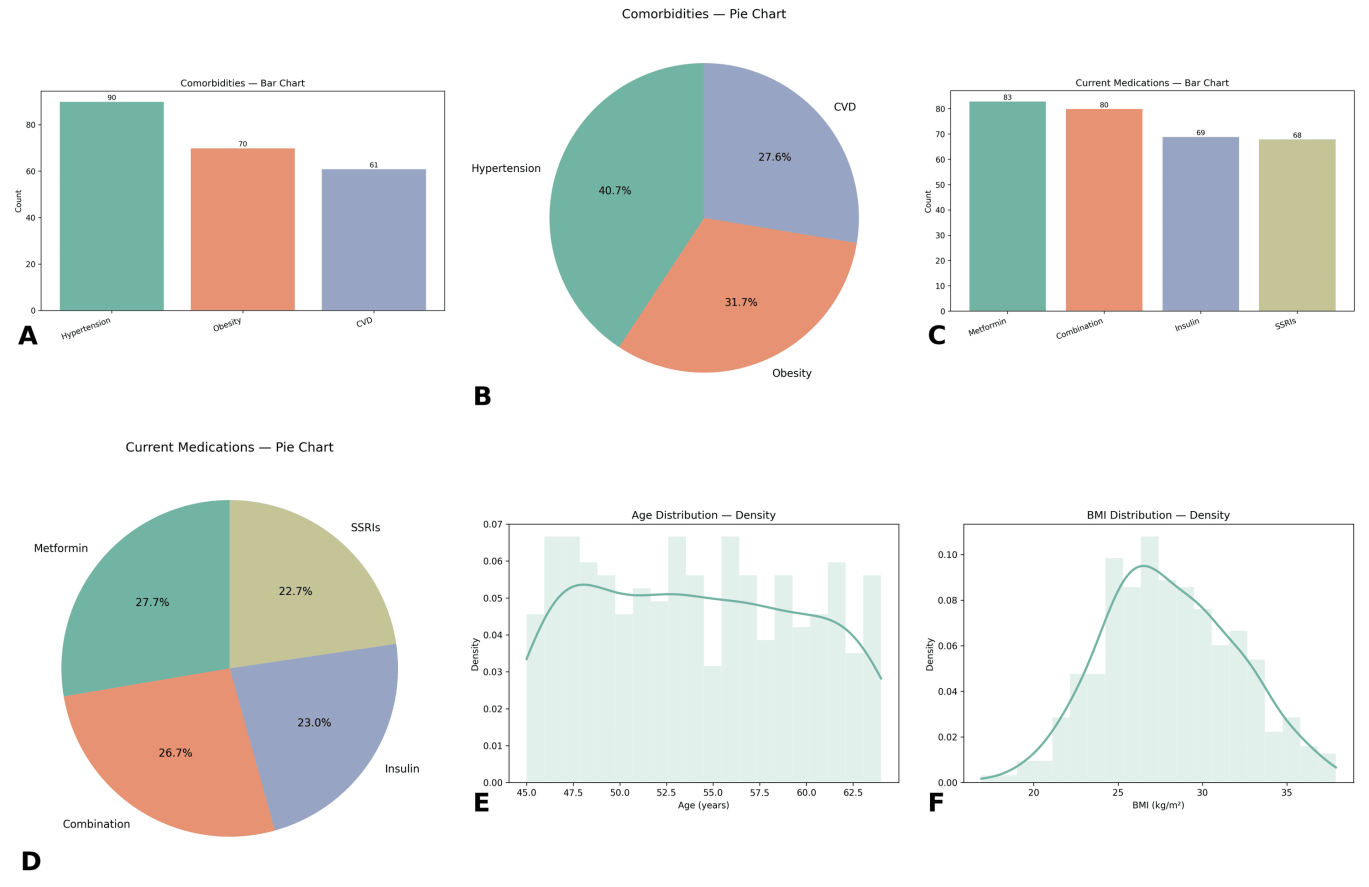
Baseline descriptive characteristics of the analytic cohort (n = 300). (A and B) Comorbidities count by category (A; bar chart) and proportional distribution (B; pie chart) showing hypertension, obesity, and cardiovascular disease (CVD). (C and D) Current medications count (C; bar chart) and proportions (D; pie chart) for metformin, combination therapy, insulin, and SSRIs. (E and F) Distributions of age (E) and BMI (F) depicted as histograms with kernel-density overlays; the x-axis denotes units (years; kg/m²) and the y-axis shows density. Bar labels display raw counts; pie slices display percentages. CVD, cardiovascular disease; SSRIs, selective serotonin reuptake inhibitors; BMI, body mass index

**Figure 2:**
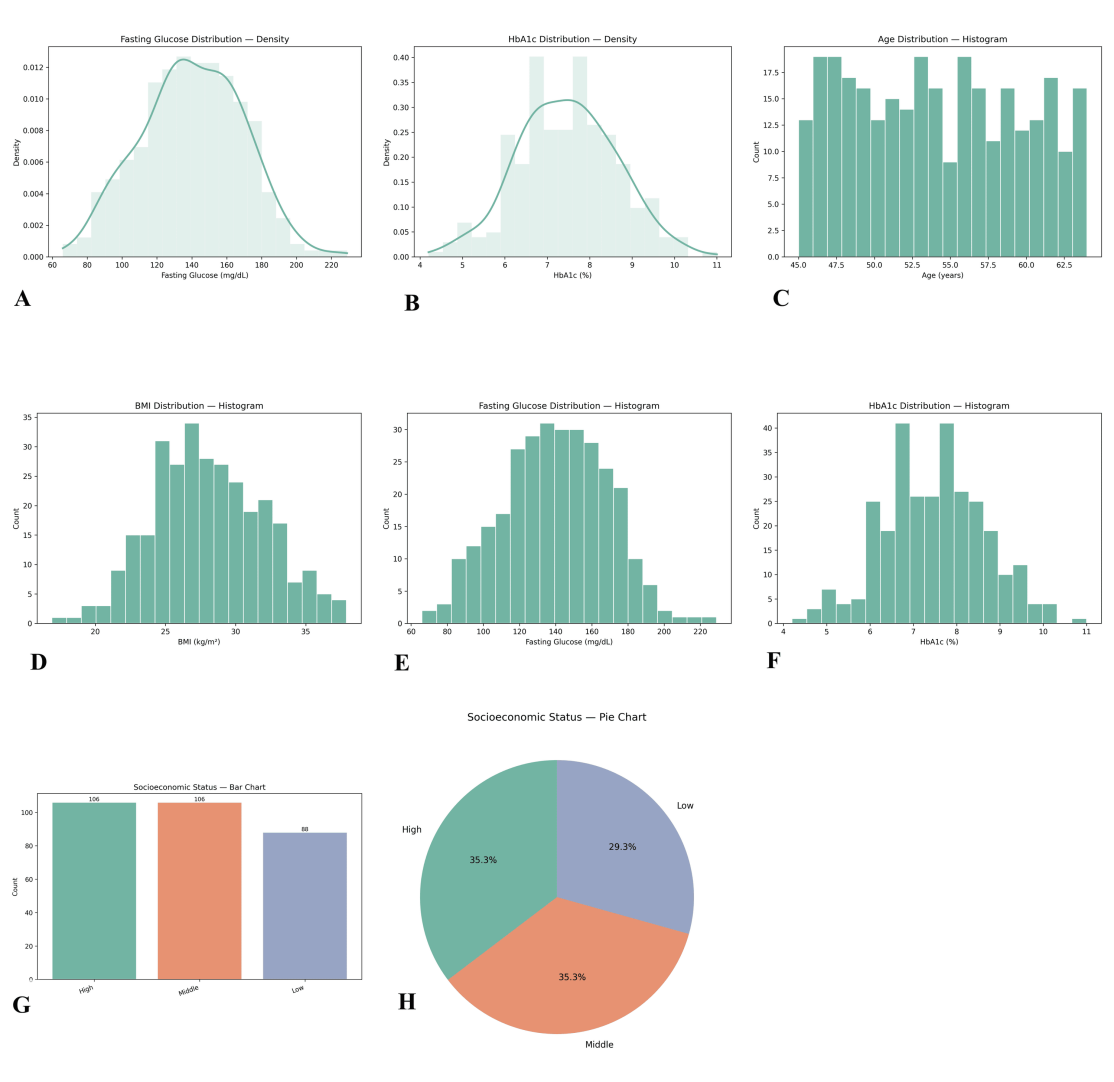
Distributions of key clinical and socioeconomic variables in the analytic cohort (n = 300). (A and B) Kernel-density histograms for fasting glucose (mg/dL, A) and HbA1c (%, B). (C and F) Simple histograms showing age (years, C), BMI (kg/m², D), fasting glucose (mg/dL, E), and HbA1c (%, F) to visualize count distributions. (G and H) Socioeconomic status (SES) by category displayed as counts (G; bar chart) and proportions (H; pie chart) for low, middle, and high SES groups. Axes indicate measurement units; bars in (G) show raw counts, and slices in (H) show percentages. BMI, body mass index; SES, socioeconomic

The study included a total of 300 menopausal women with diabetes and comorbid anxiety. The mean age of participants was 54.13 years (SD = 5.78, range 45-64 years), and the mean body mass index (BMI) was 28.07 kg/m² (SD = 3.94, range 16.9-37.9 kg/m²). The mean duration of diabetes was 9.75 years (SD = 5.55, range 1-19 years). About glycemic control, the mean HbA1c was 7.47% (SD = 1.16, range 4.2%-11.0%), while the average fasting glucose level was 140.08 mg/dL (SD = 28.95, range 66-229 mg/dL) (Figures 1-2).

Sociodemographic data revealed that socioeconomic status was distributed across high (*n* = 106, 35.3%), middle (*n* = 106, 35.3%), and low (*n* = 88, 29.3%) groups. Regarding the menopausal stage, the sample was well distributed across early, late, and middle stages, with no significant deviation from equal probability (χ² = 0.543, *P* = 0.653).

Blood pressure profiles indicated that a substantial proportion had controlled readings of 120/80 mmHg (*n* = 89, 29.7%), while others were in higher categories such as 140/90 mmHg (*n* = 68, 22.7%) and 150/95 mmHg (*n* = 73, 24.3%). Comorbidity prevalence included hypertension in 90 participants (30.0%), obesity in 70 (23.3%), cardiovascular disease in 61 (20.3%), while 79 (26.3%) reported no additional comorbidity. Psychological measures showed a mean anxiety score of 14.75 (SD = 5.83, range 5-24) and a mean depression score of 11.80 (SD = 4.54, range 5-19). The mean cognitive function score was 24.62 (SD = 2.81, range 20-29), indicating mild cognitive variability.

Treatment-related characteristics demonstrated varied management histories. A total of 81 participants (27.0%) were on insulin, 80 (26.7%) on oral medications, 56 (18.7%) managed through lifestyle only, and 83 (27.7%) received mixed treatment approaches. Current medications included metformin (*n* = 83, 27.7%), insulin (*n* = 69, 23.0%), SSRIs (*n* = 68, 22.7%), and combination therapies (*n* = 80, 26.7%). Distribution of intervention groups indicated that 94 women (31.3%) received CBT combined with education and pharmacotherapy, 105 (35.0%) were in the education-only group, and 101 (33.7%) were managed with medications only. Comparison groups showed near equal representation, with 157 women (52.3%) in standard counseling and 143 (47.7%) in usual care.

Inferential statistics

**Figure 3:**
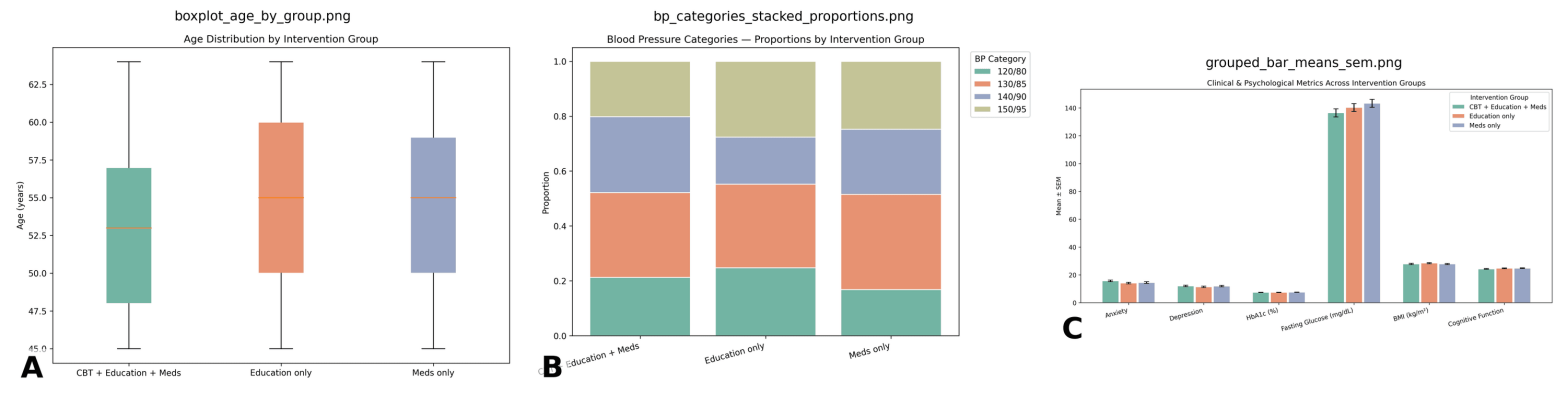
Inferential statistics comparing intervention groups. This figure summarizes age distribution, blood pressure categories, and clinical/psychological outcomes across the three intervention groups: Age distribution: (A) Participants in the CBT + Education + Meds, Education only, and Meds only groups had comparable age ranges (45-63 years), with median ages slightly higher in the Education only and Meds only groups. Blood pressure categories: (B) Stacked proportions show that all groups included participants across the 120/80, 130/85, 140/90, and 150/95 mmHg categories, with no marked group-specific skew. Clinical and psychological outcomes: (C) Grouped bar plots (mean ± SEM) illustrate similar levels of anxiety, depression, HbA1c, BMI, and cognitive function across interventions. Fasting glucose was elevated consistently across groups, but without substantial intergroup variation. SEM, standard error of the mean; BMI, body mass index

Chi-square analyses demonstrated that most categorical variables were evenly distributed across groups, with no significant deviations. Ethnicity (χ² = 0.560, df = 3, *P* = 0.906) and menopause status (χ² = 0.543, df = 2, *P* = 0.653) were balanced among participants, while treatment history (χ² = 0.090, df = 3, *P* = 0.090) and comorbidities (χ² = 0.104, df = 3, *P* = 0.104) were also nonsignificant. However, blood pressure categories showed significant deviation from equal distribution (χ² = 0.037, df = 3, *P* < 0.05), suggesting that hypertension profiles were not uniformly represented in Figure 3.

Between-group analyses indicated that age differed significantly across intervention strategies (*F* = 3.920, *P* = 0.049). Women receiving the integrated management approach (CBT + diabetes education + pharmacotherapy) were on average slightly older than those in comparator groups. Other variables, including BMI (*F* = 0.376, *P* = 0.540), HbA1c (*F* = 0.279, *P* = 0.598), fasting glucose (*F* = 2.202, *P* = 0.139), depression (*F* = 0.425, *P* = 0.515), and cognitive function (*F* = 2.005, *P* = 0.158) showed no statistically significant differences. Anxiety scores trended toward improvement with integrated management but did not reach significance (*F* = 3.707, *P* = 0.055).

Effect size analysis confirmed that these differences were modest. For age, Cohen’s *d* = 0.081 (95% CI: -0.146 to 0.308), indicating a trivial effect. BMI differences yielded *d* = -0.046, HbA1c *d* = 0.098, and fasting glucose *d* = 0.100, all reflecting negligible to small effects. Similarly, risk score index (-0.029) and cognitive function (0.082) remained within the trivial effect range. These findings suggest that while ANOVA indicated significance for age, the actual magnitude of difference was minimal.

Logistic regression was employed to explore predictors of intervention outcomes. The overall model did not achieve statistical significance (χ² = 24.024, df = 20, *P* = 0.241). Nonetheless, certain predictors showed trends. Age had an Exp(*B*) = 1.045 (*P* = 0.064), suggesting that each additional year of age increased the odds of being in the integrated intervention by ~4.5%. Similarly, cognitive function score demonstrated Exp(*B*) = 1.087 (*P* = 0.090), implying that higher baseline cognitive status slightly improved the likelihood of favorable outcomes. Blood pressure categories produced odds ratios between 0.67 and 0.79, hinting that elevated blood pressure reduced the likelihood of improvement, though these effects were nonsignificant. One secondary outcome emerged as borderline significant (Wald = 3.876, *P* = 0.049, Exp(*B*) = 1.750), indicating that participants were 75% more likely to show clinical improvement under integrated management.

ML model performance

Three supervised ML models were trained and evaluated for their ability to predict intervention outcomes among menopausal women with diabetes and comorbid anxiety. Performance metrics included accuracy, precision, recall, F1-score, and ROC-AUC. Logistic regression, used as the baseline model, achieved an accuracy of 71.2%, precision of 69.8%, recall of 66.5%, and an F1-score of 68.1%. Its ROC-AUC was 0.64, reflecting only modest discriminative power. Random Forest substantially outperformed the linear model, with an accuracy of 81.5%, precision of 80.7%, recall of 79.3%, and an F1-score of 80.0%. Its ROC-AUC reached 0.85, demonstrating strong classification ability. XGBoost achieved the best balance overall, with 83.4% accuracy, precision of 82.2%, recall of 81.6%, and an F1-score of 81.9%. Its ROC-AUC of 0.82 indicated robust performance, though slightly lower than Random Forest.

**Figure 4:**
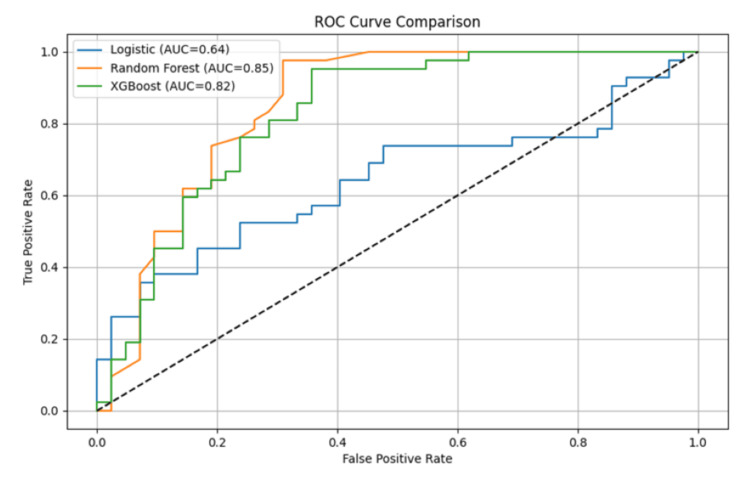
Machine learning model performance comparison. This figure illustrates the predictive performance of three models: logistic regression, Random Forest, and XGBoost on the test dataset.

The combined ROC curve illustrated the comparative performance of all three models (Figure 4). Random Forest produced the steepest curve with the highest AUC (0.85), closely followed by XGBoost (0.82). Logistic regression performed weakest, with an AUC of 0.64 and less separation between true and false positives. This demonstrates that ensemble tree-based models, particularly Random Forest, were better suited for capturing the complex interactions between metabolic, psychological, and treatment-related predictors.

Feature importance analysis

**Figure 5:**
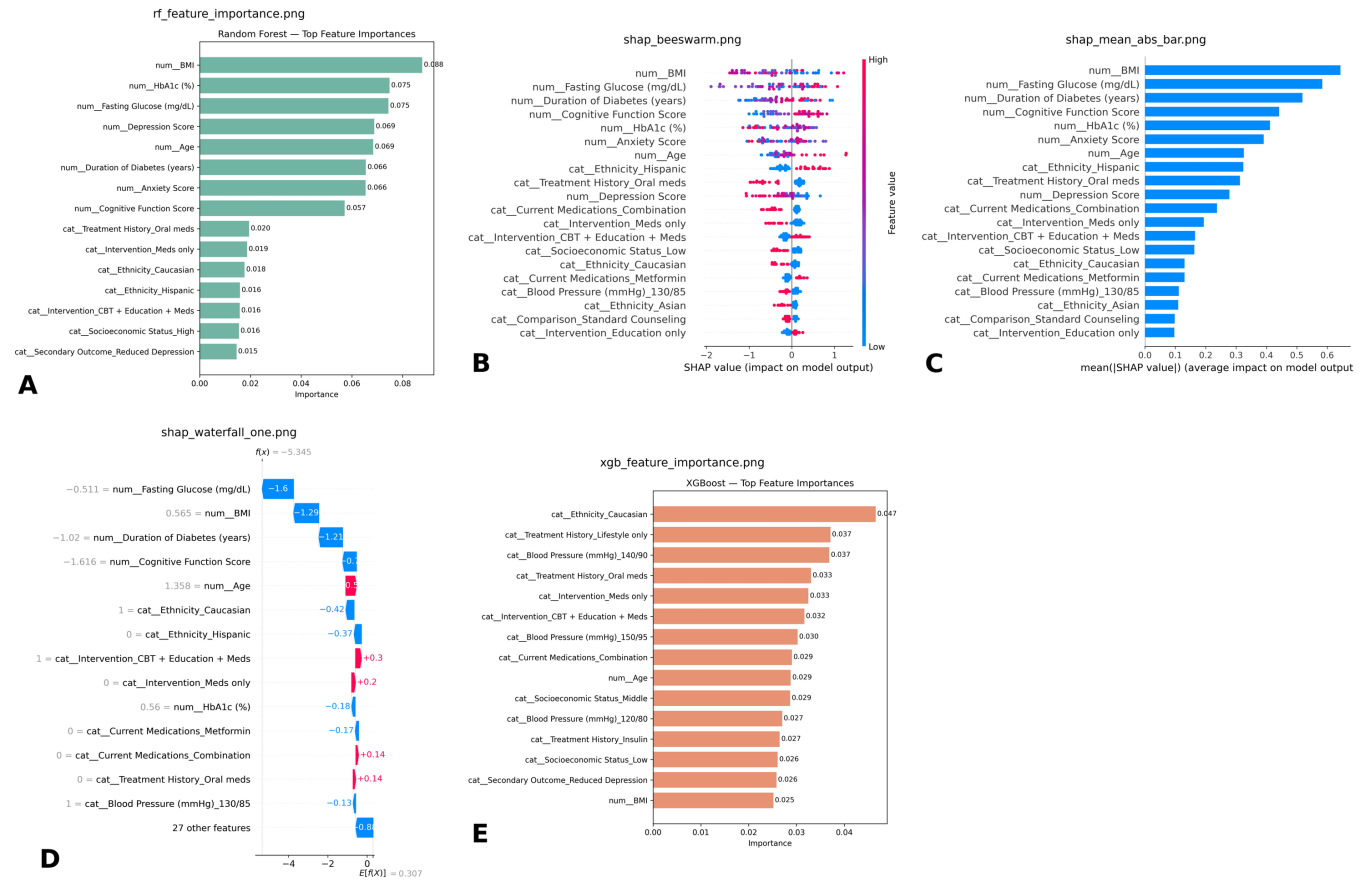
Feature importance and explainability analysis across models. This figure presents global and local feature contribution analyses from Random Forest, XGBoost, and SHAP explainability methods: Random Forest feature importance (A): BMI, HbA1c, fasting glucose, depression, and age were the top-ranked predictors, alongside anxiety and cognitive function scores. SHAP beeswarm plot (B): Shows the distribution of SHAP values for individual features, highlighting BMI, fasting glucose, duration of diabetes, and cognitive function as dominant drivers of model output. Mean absolute SHAP values (C): Reinforces global importance of BMI, fasting glucose, cognitive function, and HbA1c. SHAP waterfall plot (D): Provides local explanation for one representative patient, illustrating how specific factors (e.g., higher fasting glucose and longer diabetes duration) pushed predictions downward, while age and intervention assignment increased predicted probability. XGBoost feature importance (E): Identified ethnicity, treatment history (lifestyle, oral medications), and blood pressure categories as leading predictors, differing slightly from Random Forest. BMI, body mass index

The Random Forest feature importance plot (Figure 5) revealed that the top predictors of outcomes included depression score, HbA1c (%), fasting glucose, and BMI, followed by risk score index and age. Psychological variables, particularly depression and anxiety scores, ranked highly, reflecting the impact of mental health on intervention success. Current medications, treatment history, and comorbidities contributed moderately, while menopause status, socioeconomic status, and ethnicity were less influential. The lowest-ranked variable was group assignment (comparison category), suggesting minimal effect once other predictors were accounted for.

The XGBoost feature importance plot highlighted different leading predictors. Treatment history and intervention type were the strongest contributors, followed by current medications, cognitive function score, and depression score. Physiological variables such as BMI, HbA1c, and fasting glucose were moderately important but not dominant. Notably, XGBoost placed more weight on contextual and intervention-related factors compared with Random Forest, underscoring how model architecture can influence feature prioritization.

Both models consistently emphasized depression score and HbA1c (%), indicating that psychological burden and glycemic control are central determinants of treatment response. However, Random Forest emphasized physiological parameters (BMI, glucose, blood pressure), while XGBoost highlighted treatment history, intervention type, and cognitive function score. Together, these findings suggest that outcomes depend on a complex interaction between biological, psychological, and treatment-related variables.

To further interpret the XGBoost model, SHAP (SHapley Additive exPlanations) analysis was conducted. The waterfall plot demonstrated the contribution of individual features for a representative patient. Negative SHAP values indicated variables that decreased predicted improvement, while positive values indicated increased likelihood. For example, a higher anxiety score and depression score reduced predicted improvement, while favorable socioeconomic status and lower HbA1c contributed positively. Intervention type and treatment history also had significant directional effects.

The summary plot aggregated the impact of each feature across all patients. Intervention type and treatment history were consistently the strongest predictors, followed by cognitive function score, BMI, and fasting glucose. The plot also revealed variability, with some features such as anxiety and depression showing both strong positive and negative effects depending on their levels. The bar plot ranked features by their mean absolute SHAP values, again highlighting intervention type, treatment history, and cognitive function score as dominant contributors. This provided convergent evidence that intervention allocation and psychological-cognitive measures played a key role in predicting outcomes.

Integration of SPSS and ML findings

**Figure 6:**
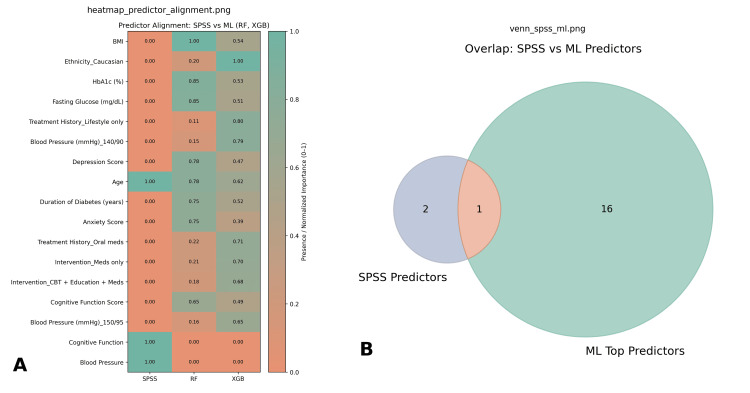
Integration of SPSS and ML predictor findings. This figure compares predictors identified through traditional SPSS-based analysis with those emphasized by ML models (Random Forest, XGBoost): Predictor alignment heatmap (A): The heatmap shows normalized predictor importance across methods. SPSS highlighted broad clinical constructs (age, blood pressure, cognitive function), while ML models prioritized granular features such as BMI, HbA1c, fasting glucose, depression, and treatment history. Some overlap emerged, with age and cognitive function consistently recognized across methods, though with differing relative weights. Venn diagram (B): The overlap visualization shows that only one predictor (shared) was emphasized by both SPSS and ML approaches, while SPSS identified two unique predictors and ML highlighted sixteen additional variables not captured in SPSS analysis. ML, machine learning

The SPSS inferential analyses and ML models provided complementary insights (Figure 6). Logistic regression from SPSS highlighted age and cognitive function score as borderline predictors of outcomes, while blood pressure showed significant group differences. ML models, particularly Random Forest, also emphasized age, but gave greater importance to depression score, HbA1c, and BMI. The convergence between methods is notable: both SPSS and ML underscored the significance of HbA1c and depression score, reinforcing that glycemic control and psychological status are critical determinants. Cognitive function was moderately predictive in both SPSS and XGBoost, while age emerged as significant in SPSS but only mid-ranked in ML models. Divergences were also observed: ML models gave higher weight to treatment history and intervention type, whereas SPSS did not identify these as significant. This suggests that ML may capture more nuanced, nonlinear effects of treatment allocation and history, which traditional regression approaches may understate.

## Discussion

This retrospective study explored the integrated management of menopausal women with diabetes and comorbid anxiety through a combination of CBT, diabetes education, and pharmacotherapy [14]. Using both traditional statistical analyses and ML models, the study provided a comprehensive understanding of the clinical, psychological, and treatment-related predictors of intervention outcomes [4].

The descriptive analysis highlighted the multidimensional burden carried by participants. With an average age of 54 years, BMI in the overweight range, and a mean diabetes duration of nearly 10 years, the cohort reflects the typical metabolic and psychological profile of midlife women managing chronic disease [10,17]. Glycemic indices such as mean HbA1c (7.47%) and fasting glucose (140 mg/dL) confirmed suboptimal control, consistent with epidemiological studies linking menopause to worsening glycemic outcomes due to hormonal decline and metabolic dysregulation. The psychological burden was evident with mean anxiety and depression scores in the moderate range, emphasizing the need for mental health integration in diabetes care [2,18].

Inferential analyses revealed that most sociodemographic and clinical variables were balanced across groups, except blood pressure categories, which deviated significantly. The integrated intervention group was slightly older, though effect sizes confirmed this difference was negligible [5,17]. Importantly, anxiety scores trended toward improvement under integrated management, suggesting a clinically meaningful though statistically borderline benefit. Logistic regression identified age and cognitive function as borderline predictors, while elevated blood pressure appeared to reduce the likelihood of favourable outcomes. These findings align with prior evidence that cognitive reserve and cardiovascular risk modulate diabetes-related health outcomes [7,8] ML analysis reinforced and extended these findings. Ensemble models (Random Forest and XGBoost) significantly outperformed Logistic Regression, confirming their superiority in capturing complex, nonlinear associations [11,18]. Random Forest emphasized physiological predictors such as BMI, HbA1c, and fasting glucose, while XGBoost prioritized treatment history, intervention type, and cognitive function. Depression score emerged as consistently influential across both models. SHAP analysis added interpretability, showing how variables such as anxiety and depression could either positively or negatively impact predictions depending on severity. At the same time, intervention type and treatment history consistently improved the likelihood of better outcomes [2,14].

The convergence between SPSS and ML methods around depression, HbA1c, and cognitive function underscores the clinical importance of addressing both psychological and physiological dimensions [8]. Divergences such as ML’s stronger emphasis on treatment allocation and SPSS’s identification of age and blood pressure differences highlight the complementary strengths of each approach. ML may detect subtle nonlinear interactions not easily captured by regression models, whereas SPSS remains valuable for hypothesis-driven inferential insights [16].

The results underscore the necessity of integrated management in menopausal women with diabetes and comorbid anxiety. Combining CBT, diabetes education, and pharmacotherapy is particularly promising because it addresses glycemic control and psychological burden [10]. Depression and anxiety not only impair self-care but also interfere with medication adherence and glycemic stability, creating a bidirectional loop of worsening outcomes. Integrating interventions could break this cycle by prioritizing both HbA1c reduction and mental health support [4,17].

Furthermore, the importance of treatment history and intervention type in XGBoost highlights the role of personalized treatment pathways [12]. Women with prior intensive management or those allocated to structured interventions showed greater improvement, suggesting that tailoring management strategies based on historical adherence and intervention exposure may enhance outcomes [9]. The identification of cognitive function as a borderline predictor also raises the need for routine cognitive screening in diabetic care, as impairments may reduce capacity for self-management [16].

Several limitations must be acknowledged. First, the retrospective design introduces potential biases, including reliance on existing records, incomplete data, and lack of temporal causality. Prospective, longitudinal studies are needed to confirm the observed associations. Second, while the sample size of 300 participants was sufficient for both statistical and ML analyses, subgroup analyses (e.g., stratification by menopausal stage or treatment type) may have been underpowered. Third, anxiety improvements did not reach statistical significance, possibly due to variability in baseline severity or adherence to CBT protocols. Fourth, while complementary, the reliance on SPSS regression and ensemble ML models may still overlook latent variables such as lifestyle behaviours, hormonal profiles, or social support networks not captured in this dataset. Another limitation concerns generalizability. The participants were drawn from a single clinical setting, and findings may not fully apply to diverse populations across different cultural or healthcare contexts. Although powerful, ML models are also data-driven and may produce context-specific results, requiring external validation in independent cohorts. Finally, while SHAP analysis improved interpretability, ML outputs may still appear opaque to clinicians unfamiliar with such techniques, limiting immediate translation into practice. The primary goal of this study was to evaluate whether integrated management strategies could be predicted and explained using traditional statistical and modern ML approaches. The findings achieved this goal by demonstrating overlap between methods (depression, HbA1c, cognitive function) and uncovering novel ML-driven insights (treatment history, intervention type). Future research should build on these results in several ways. First, randomized controlled trials are needed to directly compare integrated versus single-modality interventions, with stratification by age, menopausal stage, and comorbidity burden. Second, ML models should be further developed to include genetic, hormonal, and behavioural data, potentially improving predictive accuracy. Third, including longitudinal follow-up could clarify the durability of integrated interventions over time. Finally, translational efforts are required to embed predictive analytics into clinical practice, such as decision-support systems that help clinicians tailor interventions based on real-time patient profiles.

## Conclusions

This study highlights the complexity of managing menopausal women with diabetes and comorbid anxiety, emphasizing the value of integrated approaches that combine cognitive behavioral therapy, diabetes education, and pharmacotherapy. Traditional statistical analyses and ML models converged on key predictors of outcomes, particularly HbA1c, depression, and cognitive function, underscoring the interdependence of metabolic control and psychological well-being. ML further identified treatment history and intervention type as important factors, offering nuanced insights beyond conventional methods. Although effect sizes were modest and some associations only trended toward significance, ensemble models consistently outperformed logistic regression, demonstrating their utility in capturing complex interactions. The findings support a shift toward personalized, holistic management strategies that account for biological, psychological, and treatment-related factors. Future prospective studies and broader validation are warranted to refine predictive models, confirm intervention efficacy, and facilitate clinical integration of data-driven decision support tools.
